# Reverse genetics in high throughput: rapid generation of complete negative strand RNA virus cDNA clones and recombinant viruses thereof

**DOI:** 10.1038/srep23887

**Published:** 2016-04-05

**Authors:** T. Nolden, F. Pfaff, S. Nemitz, C. M. Freuling, D. Höper, T. Müller, Stefan Finke

**Affiliations:** 1 Friedrich-Loeffler-Institut, Federal Research Institute for Animal Health, Institute of Molecular Virology and Cell Biology, D-17493 Greifswald – Insel Riems, Germany; 2 Friedrich-Loeffler-Institut, Federal Research Institute for Animal Health, Institute of Diagnostic Virology, D-17493 Greifswald – Insel Riems, Germany

## Abstract

Reverse genetics approaches are indispensable tools for proof of concepts in virus replication and pathogenesis. For negative strand RNA viruses (NSVs) the limited number of infectious cDNA clones represents a bottleneck as clones are often generated from cell culture adapted or attenuated viruses, with limited potential for pathogenesis research. We developed a system in which cDNA copies of complete NSV genomes were directly cloned into reverse genetics vectors by linear-to-linear RedE/T recombination. Rapid cloning of multiple rabies virus (RABV) full length genomes and identification of clones identical to field virus consensus sequence confirmed the approache’s reliability. Recombinant viruses were recovered from field virus cDNA clones. Similar growth kinetics of parental and recombinant viruses, preservation of field virus characters in cell type specific replication and virulence in the mouse model were confirmed. Reduced titers after reporter gene insertion indicated that the low level of field virus replication is affected by gene insertions. The flexibility of the strategy was demonstrated by cloning multiple copies of an orthobunyavirus L genome segment. This important step in reverse genetics technology development opens novel avenues for the analysis of virus variability combined with phenotypical characterization of recombinant viruses at a clonal level.

More than twenty years after the first recovery of recombinant RABV from cDNA^1^ reverse genetics technologies for negative strand RNA viruses (NSVs) significantly accelerated virus research as targeted mutagenesis of NSV genomes allowed direct proof of concepts about virus replication and pathogenesis. The possibility of genetic manipulation also allowed development of novel vector vaccines against zoonotic and emerging diseases like RABV^2^, Ebola virus^3^, Lassa^4^, Henipa-^5^ and Human Immunodeficiency viruses^6^. Reporter gene expressing virus vectors have been developed to map trans-synaptic connections between neurons^7^,^8^ or to follow virus tropism in infected hosts^9^,^10^.

From the beginning, reverse genetics technologies have been constantly improved. For instance, vaccinia virus free T7 polymerase rescue systems^11^, use of T7 polymerase independent systems^12^,^13^,^14^,^15^ and optimized ribozyme sequences flanking the virus cDNA in reverse genetics vectors^16^ substantially increased the efficiency of many NSV rescue systems.

Whereas optimized recovery systems allowed easy and rapid generation of recombinant viruses, the availability of only a limited number of cloned infectious full-length cDNA genomes is still a bottleneck. Many of the broadly used cDNA clones have been generated from cell culture adapted or attenuated vaccine strains^1^,^17^,^18^ and limitations in usability for pathogenesis research exist. In case of RABV, gene dependent variations in pathogenesis were studied by insertion of heterologous sequences in attenuated vaccine backbones^19^,^20^,^21^. However, only fully virulent virus backbones allow assessment of the real contribution of targeted gene functions to virus replication and disease development *in vivo*. So far, recombinant silver haired bat lyssavirus^22^ and European bat lyssavirus type 1^12^ are the only published examples of recombinant lyssaviruses derived from virulent progenitors that have not been adapted to cell cultures or mouse models. As both viruses are of bat origin and although silver haired bat lyssavirus is known to cause lethal infection in bats and men^23^, tools to investigate host species dependent mechanisms of virus replication and maintenance in reservoir species like fox and dog are limited due to the lack of suitable recombinant virus systems.

In view of rapidly increasing information on sequence variability within virus populations from modern deep sequencing technologies^24^, efficient cloning of the relevant virus genomes in reverse genetics vectors for targeted genetic modification or verification of mutations in individual genome copies is an important step towards combining large scale sequence analysis with the power of reverse genetics. A rapid and flexible cloning system for full-length infectious cDNA virus clones from NSVs is therefore a goal that still has not been satisfactorily achieved.

RedE/T-cloning, also known as RedE/T-recombineering, is a convenient way to manipulate molecules such as Bacteria-, Yeast-, and P1-derived Artificial Chromosome vectors (BAC, YAC, PAC) or even *E. coli* chromosomes through the Rac- or Lambda- phage protein pair RecE/RecT or Redα/Redβ respectively^25^,^26^. Originally, recombineering depended on an actively replicating circular backbone such as BAC, PAC or YAC vectors in which a linear DNA fragment containing a selection marker is integrated by linear-to-circular homologous recombination (LCHR), when the fragment is flanked by short sequences with homology to the target vector (Fig. 1A). In an ingenious approach RecE/RecT-based cloning strategies were further improved by recombination between two linear DNA fragments (LLHR) of which one was a PCR-amplified minimal vector backbone flanked by primer derived homology arms^26^,^27^. This new technology allowed specific and highly efficient insertion of linear DNA molecules into linearized minimal *E. coli* plasmid vectors (Fig. 1B).

Here, we adapted linear-to-linear recE/recT-mediated homologous recombination (LLHR) in *E. coli* for fast and efficient cloning of complete RABV genome cDNAs into reverse genetics plasmid vectors (Fig. 1C). Efficient cloning of genome cDNAs from different street isolates, successful recovery of recombinant RABVs and generation of reporter gene expressing viruses illustrate the power of our new strategy. Beyond rapid generation of virus cDNA clones, the high efficiency of the recombinatorial full genome cDNA cloning allowed sequence analysis on a clonal level and correlation with deep sequence analyses of the parental field viruses. Moreover, the flexibility of the system was demonstrated by fast translation of the protocol to the efficient cloning of segmented orthobunyavirus L cDNA.

## Results

### Generation of full length cDNA clones by linear-to-linear homologous recombination (LLHR) in *E.* coli

Prior to full length cDNA amplification and recombinatorial insertion in plasmid vectors, the 3′ and 5′-terminal sequences of two different RABVs of dog and fox origin and of the attenuated lab strain SAD L16 as a control were determined by vRNA genome end-joining and RT-PCR sequencing (Supplementary Fig. S1). The terminal fox and dog RABV sequences differed from SAD L16 by 9 and 8 nts (3′-terminal leader) and 9 and 11 nts (5′-terminal trailer), respectively (Supplementary Fig. S1). These data confirmed the expected sequence variability between the virus isolates and provided sequence information for the design of virus isolate specific homology arms for recE/recT-mediated recombination. The homology arms of primers (Table 1) consisted of the terminal 50 nts of the genome ends, followed by target vector sequences for PCR amplification of the linear minimal vector DNA.

**Figure 2:**
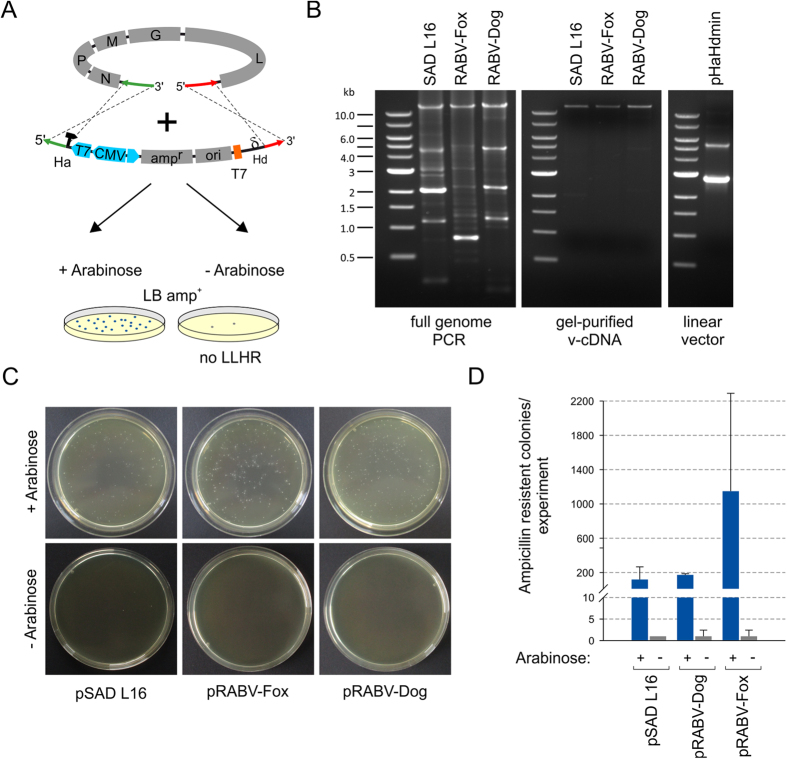
LLHR of virus genomic cDNA and pHaHdmin linear vector DNA. Schematic presentation of the performed LLHR experiment. Linear full length genome PCR products and PCR-amplified linear vector DNA were electroporated into into arabinose induce or non-induced E. coli Gb05-Dir. (**A**). Agarose gels showing full genome PCR products, gel purified 12 kb PCR products and PCR-amplified linear minimal cloning vector pHaHdmin (**B**). The effectiveness of LLHR in the presence of arabinose was quantified by colony counting after selection on ampicillin containing LB-plates (LB amp^+^) (**C**). The mean of absolute colony numbers from three independent recombination experiments are shown with indicated standard deviation by error bars (**D**).

For insertion of full length cDNA copies into a plasmid vector by LLHR a derivative of pHaHd MiniGFP^12^ was used in which non-essential vector sequences were deleted to minimize the risk of intramolecular recombination (Supplementary Fig. S2). The minimal vector pHaHdmin was used as a template for PCR amplification of a linear 2.7 kb vector DNA with primers comprising the virus isolate specific 50 nt homology arms (Table 1 and Supplementary Fig. S2). Genomic RABV RNA from infected cell monolayers was reverse transcribed by SuperscriptIII reverse transcriptase with primers (Supplementary Table S3) binding to the first 18 nts of the leader RNA coding sequence. Full-genome PCRs were performed with the same forward primer as used for cDNA synthesis and an appropriate reverse primer (Supplementary Table S3). For all tested RABVs an 11.9 kb full length cDNA was successfully generated (Fig. 2B).

PCR amplified linear full length virus genome and vector DNA comprising the virus-specific homology arms were gel purified and co-electroporated into *E. coli* GbDir-05 (Fig. 2A). Arabinose-controlled induction of recE/recT before electroporation resulted in the selection of multiple ampicillin resistant *E. coli* clones, whereas only few colonies were observed in the absence of induction. Recombination efficiencies were calculated by colony counting of arabinose induced vs. not-induced recombination reactions. The recE/recT dependent 100 to 500 fold increase in colony numbers with absolute clone numbers in a range of 200 and 1000 colonies per electroporation indicated that the recombination was highly effective (Fig. 2C,D).

**Figure 3:**
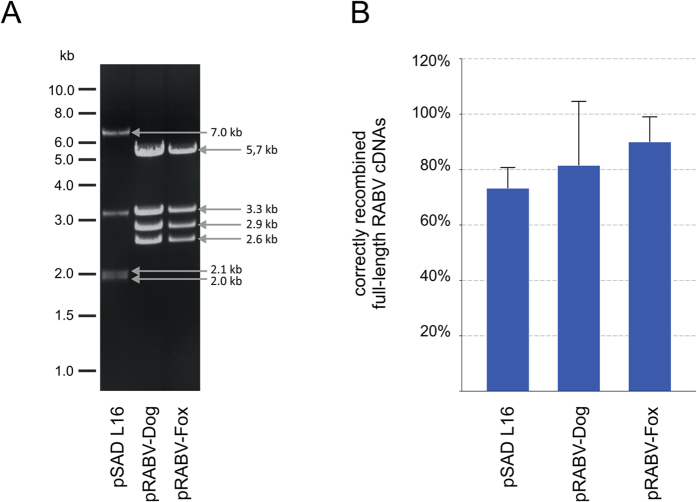
*Sac*I digest of ampicillin resistant recombinants revealed that LLHR of full length RABV cDNAs occurs at high frequency. (**A**) Exemplary restriction pattern of pSADL16, pRABV-Dog and pRABV-Fox full length cDNA plasmids after SacI digest. A virus cDNA clone was considered positive, when the sum of all fragment sizes corresponded to the expected 14.5 kb plasmid (11.9 kb virus genome plus 2.7 kb vector fragment). (**B**) Correctly recombined full length RABV genomes were averaged from two independent LLHR experiments and expressed as percentage of analyzed recombinants.

To test whether complete full genome virus sequences have been inserted, plasmids were purified from *E. coli* clones and the presence of the expected 11.9 kb insert and the 2.7 kb vector backbone was monitored by *Sac*I restriction endonuclease digests (Fig. 3A and Supplementary Fig. S4). Remarkably, 73 to 88% of a minimum of 15 tested clones per replicate contained the insert of 11.9 kb (Fig. 3B).

### Sequence analysis of individual full length cDNA clones by deep sequencing

To compare the cloned full length cDNA copies with sequences of the progenitor virus isolates, the complete consensus sequences of the parental RABV isolates from fox (Germany, 1998; Accession#: LN879481) and dog (Azerbaijan, 2002; Accession#: LN879480) were determined by ion torrent sequencing. With 4 and 3 positions in the wt RABV-Fox and wt RABV-Dog isolates, the number of single nucleotide polymorphisms was remarkably low (Table 2).

Four of the fox and four of the dog RABV-derived cDNA clones were randomly selected and also analyzed by ion torrent sequencing. The pRABV-Fox clones 8, 9, 10 and 11 differed from the progenitor sequences by 3, 4, 3 and 5 single nucleotide exchanges, respectively (Table 3A) resulting in nucleotide identities above 99.9% for all tested clones. Only one amino acid exchange was detected in the nucleoprotein N of clones 8 and 9 (M60T and V23A, respectively). In clone 11 four amino acid exchanges in the matrix protein M (P45S) and in the polymerase L protein (I140V, V1183I and E1522Q) were detected, whereas no alteration was observed in clone 10. Non-silent mutations in fox clones 8, 9 and 11 were absent from the parental virus population (Table 2), indicating that either the mutations arose from erroneous RT or inaccurate full-genome amplification before LLHR or that those mutations represent a minor fraction in the progenitor virus that has not been detected by the deep sequencing analysis. Notably, a silent single nucleotide polymorphism (SNP) in the G gene at nt position 4015 (76% C and 23% T) identified in the progenitor virus was reflected by the detection of a C residue in clones 8 and 11 and of a T residue in clones 9 and 10.

Of the four pRABV-Dog clones 3, 5, 14 and 15 only two contained nucleotide alterations compared to the progenitor virus (Table 3B). Whereas one silent mutation was found in clone 15, clone 3 contained one silent mutation, two amino acid exchanges in G (F14S, L451P) and one amino acid exchange in L (A408V). These data showed that the cloned full length genomes exhibited a remarkably low level of variability, although few SNPs were detectable. Recovery of individual SNPs preexisting in the progenitor virus sequence in individual pRABV full length clones (see fox isolate position 4015) indicated that efficient cloning of full length cDNAs into plasmid vectors allows confirmation of naturally occurring SNPs detected by modern deep sequencing approaches. Moreover, combinations of SNPs can be detected by the full length clone analysis.

### Rescue of recombined RABV from cloned cDNAs

A major benefit of the new method is the direct recovery of infectious virus clones from the individual vectors. This not only allows sequence analysis of individual genome molecules but also functional downstream analysis of selected mutants and further targeted modification of newly cloned virus isolates.

We therefore checked whether infectious viruses could be recovered from the eight full genome cDNA encoding plasmids described above. Virus rescue was supported by expression plasmids for the N, P and L proteins of SAD L16^29^. Although recovery of authentic recombinant fox and dog street RABVs was expected to be less efficient than of cell culture adapted SAD L16, rescue of the fox and dog virus clones occurred at a remarkably high rate (Table 4). Virus was successfully rescued from dog RABV full length clones 5, 14 and 15 and fox RABV clones 9 and 10 in three independent experiments. One of three attempts with clone 3 (rRABV-Dog) and two of three trials with clone 11 (rRABV-Fox) were successful, whereas clone 8 from fox RABV failed to yield progeny virus. In this clone, sequence analysis revealed a single nucleotide deletion at position 3192 which resulted in the formation of a transcription stop signal like 7 U stretch, positioned 79 nts upstream of the M mRNA transcription stop signal.

After passage of cell culture supernatants from transfected BSR-T7/5 cells to Na42/13 cells, infection and subsequent virus spread was monitored by indirect immunofluorescence against RABV N protein (not shown). Detection of N positive infected foci for rRABV-Fox clones 9 and 10 as well as for rRABV-Dog clones 5, 14 and 15 confirmed rescue of recombinant virus from the cloned cDNAs. In contrast, rRABV-Fox clone 11 as well as rRABV-Dog clone 3 were not detected in the passage experiments, although staining for virus expressed G protein in the transfected BSR T7/5 cells indicated successful virus rescue (Table 4). In rRABV-Fox clone 11 and rRABV-Dog clone 3 non-silent mutations in L may have affected the ability to replicate in the absence of plasmid expressed P and L (see Table 2).

### Generation of reporter expressing rRABV-Dog

Reporter protein expressing viruses represent important tools to investigate tropism and pathogenesis of viruses. Therefore, we tested whether non-fixed virulent RABV with limited virus replication in cell culture tolerate insertion and expression of a reporter gene from an extra cistron.

**Figure 4:**
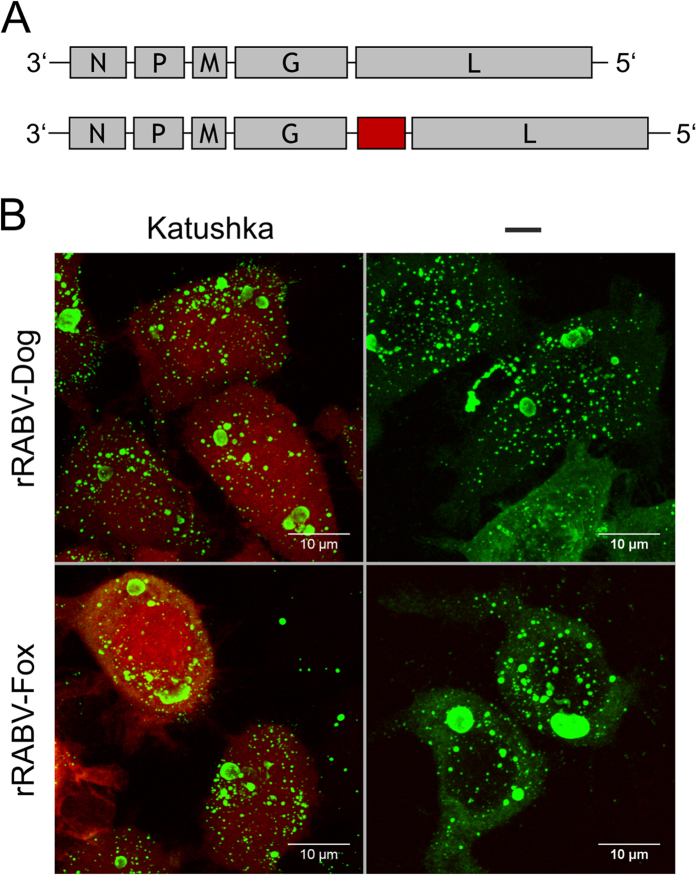
Red fluorescent protein expression from recombinant dog and fox viruses. (**A**) Genome organization recombinant viruses. The red fluorescent Katushka reporter gene was inserted between the G and L genes. (**B**) Red fluorescence in virus infected cells and indirect immunofluorescences against RABV N (green) in N42/13 cells at 48 hrs post infection. Left: rRABV-Dog-Katushka (top) and rRABV-Fox-Katushka (bottom). Right: rRABV-Dog clone 14 (top) and rRABV-Fox clone 9 (bottom). Shown are maximum- projections of confocal z-stacks.

After insertion of a multiple cloning site upstream of the G/L gene border, the red fluorescent Katushka reporter gene together with the conserved gene border sequence from SAD L16 was inserted in pRABV-Dog 14 (Supplementary Fig. S3) and pRABV-Fox 9. Recombinant rRABVs expressing the reporter gene were successfully recovered from cDNA and virus spread on Na42/13 cells was monitored by red fluorescence. As demonstrated by confocal laser scan microscopy (Fig. 4), the red fluorescent reporter was expressed both in rRABV-Dog Katushka and rRABV-Fox Katushka infected cells, whereas no red fluorescence was observed in rRABV-Dog and rRABV-Fox infected cells.

These data demonstrated that even in a non-cell culture adapted genetic background insertion of additional transcription units and successful recovery of replicating virus is possible.

### Growth kinetics of parental and recombinant RABV

**Figure 5:**
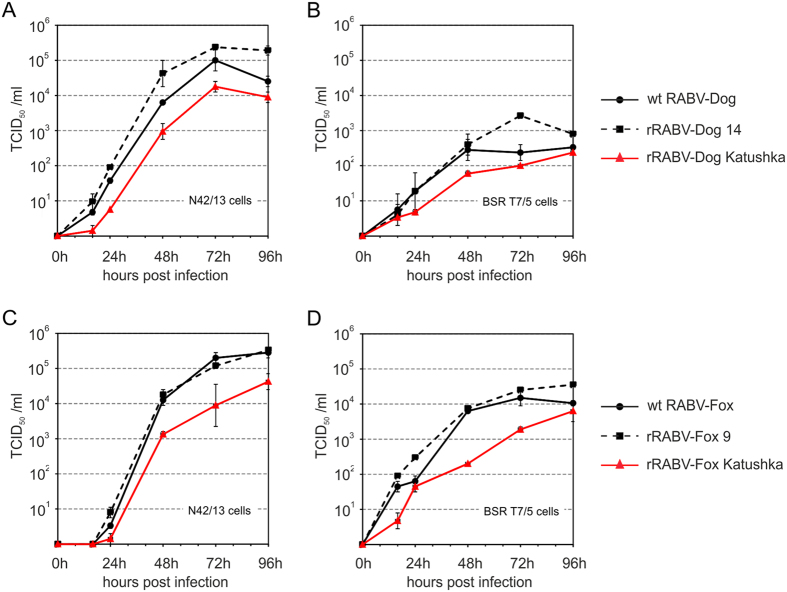
Growth kinetics of recombinant RABVs and wild type field isolates from fox and dog. N42/13 neuroblastoma and BSR T7/5 cells were infected with a MOI of 0.01 ffu/ml and samples were taken at 0, 16, 24, 48, 72 and 96 hours post infection (hpi) and infectious virus titers were determined on N42/13 cells. (**A**) wt RABV-Dog, rRABV-dog clone 14 and rRABV-Dog Katushka on N42/13 neuroblastoma cells. (**B**) wt RABV-Dog, rRABV-dog clone 14 and rRABV-Dog Katushka on BSR T7/5 baby hamster kidney cells. (**C**) wt Fox-Dog, rRABV-fox clone 9 and rRABV-Fox Katushka on N42/13 neuroblastoma cells. (**D**) wt Fox-Dog, rRABV-fox clone 9 and rRABV-Fox Katushka on BSR T7/5 baby hamster kidney cells. Error bars indicate min/max values from two indepentend experiments.

Comparative growth curves revealed that both, replication kinetics of rRABV-Dog clone 14 and rRABV-Fox clone 9, were comparable to their parental virus isolates (Fig. 5A,C), with maximum titres of 10^5^ and 10^5.4^ TCID_50_/ml for wt RABV-Dog and rRABV-Dog clone 14 on N42/13 neuroblastoma cells (Fig. 5A) and 2.0 and 1.5 logs fold lower titres on non-neuronal BSR T7/5 cells widely used for efficient amplification of RABV laboratory strains. These experiment showed that virus production on those cell types and in particular the typical field isolate specific requirement of neuroblastoma cells for efficient virus replication, was preserved in the recombinant virus.

The titres of rRABV-Dog Katushka remained about 1 log below the wt RABV-Dog and rRABV-Dog clone 14 titres, indicating an attenuating effect of the extra gene expression on rRABV-Dog replication. In contrast, replication on BSR T7/5 cells was decreased comparable to wt RABV-Dog and rRABV-Dog clone 14, indicating that the extra gene insertion did affect virus replication in general, but did not affect field virus isolate specific replication in neuronal cells.

With maximum titer of 10^5^ and 10^5.5^ TCID_50_/ml on N42/13 cells, wt RABV-Fox and rRABV-fox clone 9 (Fig. 5C) were not only comparable to each other, but also exhibited similar replication kinetics than wt RABV-Dog and rRABV-Dog clone 14. However, with a 1.3 and 1.2 logs fold drop in BSR T7/5 cell specific titres (Fig. 5D) the specific infectivity for the non-neuronal cell line was higher than observed for wt RABV-Dog and rRABV-Dog clone 14. As observed for the dog viruses, insertion of a reporter gene into the rRABV-Fox genome let to decreased titres on both, N42/13 and BSR T7/5 cells.

### Virulence of selected recombinant clones in mice

To assess whether the recombinant virus clones were also *in vivo* comparable to their parental field virus isolates, the ability to cause disease in 3 to 4 week old BALB/c mice was monitored after inoculation by the i.c. and i.m. routes.

**Figure 6:**
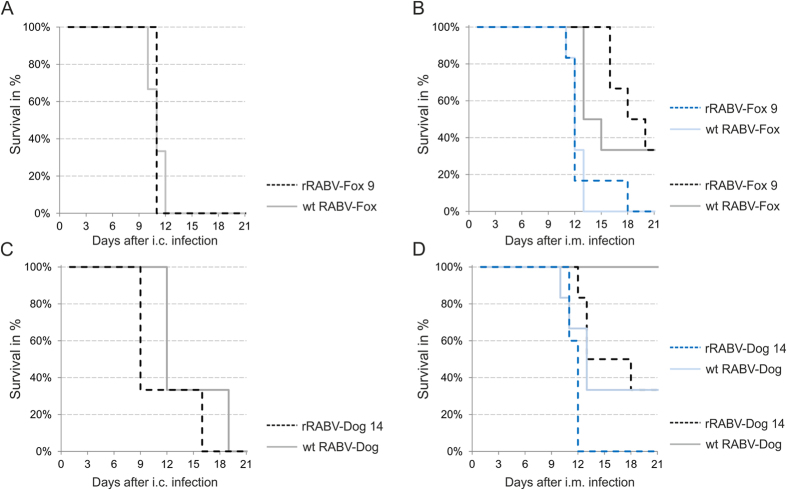
Virulence of progenitor fox and dog virus isolates and selected recombinant clones rRABV-dog clone 14 and rRABV-fox clone 9 after intracranial (i.c.) and intramuscular (i.m.) inoculation of 3 to 4 week old BALB/c mice. Groups of 5 animals were inoculated with the indicated viruses either by the i.c. (50 ffu/animal) or by the i.m. (5000 or 50 ffu/animal) route of infection. Survival of infected mice was monitored daily. (**A**) Survival of mice after i.c. inoculation with wt rRABV-Fox virus and recombinant rRABV-Fox clone 9. (**B**) Survival of mice after i.m. inoculation with wt rRABV-Fox virus and recombinant rRABV-Fox clone 9 at doses of 5000 ffu/animal (blue lines) and 50 ffu/animal (grey/black lines). (**C**) Survival of mice after i.c. inoculation with wt rRABV-Fox virus and recombinant rRABV-Fox clone 9. (**D**) Survival of mice after i.m. inoculation with wt rRABV-Fox virus and recombinant rRABV-Fox clone 9 at doses of 5000 ffu/animal (blue lines) and 50 ffu/animal (grey/black lines).

All mice inoculated by the i.c. route of infection with 50 ffu of rRABV-Fox clone 9, wt RABV-Fox, rRABV-Dog 14 and wt RABV-Dog developed clinical signs and were euthanized after reaching a clinical score of 3. Whereas both, mice infected with rRABV-Fox clone 9 and with wt RABV-Fox survived no longer than 11 days (Fig. 6A), survival time of the rRABV-Dog clone 14 and wt RABV-Dog infected mice was prolonged up to 16 and 19 days, respectively (Fig. 6C). These data indicated, that the clinical course of disease was different for the fox and dog virus isolates and that in i.c. infections those differences was preserved in the recombinant viruses.

Most importantly, both recombinant virus clones were able to cause disease after i.m. inoculation at higher and low virus doses (5000 and 50 ffu/ml). Similar survival times of rRABV-Fox clone 9 and wt RABV-Fox after high dose infection indicated, that these viruses were comparable in peripheral infections. After low dose i.m. infections with both viruses, 33% of the animals survived (Fig. 6B), confirming comparable virulence of the wt field isolated and the recombinant clone 9. However, three to 4 days prolonged survival times for rRABV-Fox clone 9 may also indicate minor differences that could be caused by the clonal genetic background.

In contrast to the fox viruses, rRABV-Dog clone 14 and wt RABV-Dog differed in i.m. inoculations (Fig. 6D). Whereas all mice established lethal rabies disease after high dose rRABV-Dog clone 14 infection, 33% of wt RABV-Dog infected mice survived. After low dose infection, all mice infected with the wt RABV-Dog isolate survived, whereas rRABV-Dog clone 14 caused disease in 66% of infected animals. These data showed that rRABV-Dog clone 14 was better infecting mice from the periphery than the progenitor virus isolate.

Overall, the *in vivo* data demonstrated that the virulence of the clonal recombinant virus rRABV-Fox clone 9 and rRABV-Dog clone 14 did not loose their ability to cause rabies disease in the mouse model after peripheral inoculation, even at low dose infections.

### Detection of selected mutations after infection in heterologous carnivore host

The power of the new methodology was further assessed by analysis of clonal virus genomes after passage through a heterologous carnivore host. From brains of raccoons that have been experimentally infected by the intramuscular route with RABV-Fox^30^, virus was re-isolated on Na42/13 cells and RNA was prepared for full length genome RT-PCR and subsequent LLHR in *E. coli*. After selection of 160 ampicillin resistant *E. coli* colonies, 16 clones were used for identification of full-length genome cDNA insertion by restriction endonuclease digest (not shown). Identification of 12 positive rRABV-Fox(rac) clones confirmed the high efficiency of the method (Figs 2 and 3). Four of the rRABV-Fox(rac) clones were randomly selected and sequenced by next-generation sequencing (NGS). The whole-genome sequences were aligned to the consensus sequence of the progenitor fox isolate. At the nucleotide level 4, 4, 2 and 4 nt replacements for rRABV-Fox(rac) clones 1, 2, 3 and 4 were detected (Table 5). Interestingly, replication in the raccoon host resulted in the selection of amino acid replacements in G and L, whereas no amino acid substitutions were observed in N, P, and M. Whereas 3 amino acid alterations in L (V382I in clone 2 and T118A/W925R in clone 4) and one in G (K458T in clone 1) were clone specific, two amino acid exchanges (D266N and K508T) were detected in all four cloned virus genomes. Of them, the non-silent mutation D266N was already present in the progenitor virus population, whereas K508T was not detectable in the progenitor virus deep sequence, at least beyond the thresholf of 5% frequency (Table 2). Blast search and comparison of 999 G sequences revealed that the amino acids D and N were present in 88% and 8% of the G sequences, respectively. In contrast, with 56% K and 54% R at position 508 in the cytoplasmic domain of G, a positively charged amino acid was perfectly conserved in all sequences and the identified exchange to threonine obviously was not pre-formed in natural viruses. These data show that fast and efficient cloning of complete virus genomes not only allows detection of possible adaptive mutations after bottleneck infections but also allows identification of different combinations of single site mutations in individual genomes.

### Flexibility of the system - application to other viruses

Finally, we tested whether the new cloning strategy is applicable to other NSVs. This included the complete workflow of end sequence determination, long range RT-PCR and subsequent cloning into a reverse genetics plasmid vector that contained a virus-specific hammerhead ribozyme sequence.

To this end, genome segment RNAs from the orthobunyavirus Batai Virus (BATV; isolate South West Germany, 2009^31^) were ligated and the terminal sequences were determined by RT-PCR sequencing (not shown). Next, PCR primers for RT-PCR amplification of the complete 6.9 kb L genome segment of the virus and for PCR amplification of the linear minimal vector pHaHdmin for LLHR were designed. The latter comprised the 50 nt homology arms and pHaHdmin-specific sequences. Additionally, the oligonucleotide BATV Le-rev (Supplementary Table S3) covers not only the 50 nt of the BATV leader sequences but also the region of the hammerhead ribozyme in front and replaces the RABV-specific 9 nucleotides to a BATV-specific hammerhead ribozyme.

After LLHR of the PCR-amplified L-segment with the pHaHdmin-derived linear vector in *E. coli*, 100% of the analyzed ampicillin resistant *E. coli* colonies were tested positive for the insertion of the genomic BATV L segment cDNA (not shown). Sequencing of two randomly picked recombinants revealed that both BATV L recombinants were 100% identical to each other. Direct RT-PCR sequencing of two independent RT-PCR products from virus RNA perfectly confirmed the sequences obtained in the clones, and excluded any randomely introduced sequence errors in the course of recombinatorial cloning. Accordingly, our data showed that the L segment of the here used progenitor virus (BATV; isolate South West Germany, 2009) differed from a closely related Italian BATV isolate (Accession: KC168048) by five silent nucleotide exchanges.

These data demonstrated that LLHR works independently of the complete virus genome sequence, and adaption of the functional elements in the vector backbone of pHaHdmin flanking the recombineered viral cDNA to other NSV families can be easily done.

## Discussion

We present a novel strategy for fast cloning of complete infectious negative strand RNA virus genomes into reverse genetics plasmid vectors. Beyond cloning of genomic virus cDNAs in a restriction endonuclease independent manner, the novel system exhibits several benefits for cloning and generation of recombinant negative strand RNA viruses that could create a milestone in reverse genetics technology: (i) Cloned genomes in the full length cDNA plasmid are derived from genome copies that really exist in the authentic progenitor virus populations, artificial combinations of sub-fragments can be excluded. (ii) The high efficiency of full length cDNA cloning opens novel possibilities to combine modern NGS methods of sequence analysis with the assessment of sequence variation in individual genome copies. (iii) Direct recovery of individual virus mutants allows rapid experimental proof of concepts generated by sequence analysis. (iv) Customized recombinant viruses from relevant reservoir hosts are easy to generate and will advance future pathogenesis research. (v) The flexibility by using PCR primers in the system allows quick adaptation of reverse genetics vectors to phylogenetically distant viruses.

Although RecE/T-mediated homologous recombination in *E. coli* has been used mainly for genetic modification of large DNA fragments such as BACs, YACs or even *E. coli* chromosomes^26^,^27^, efficient direct insertion of full length cDNA genome copies of RNA viruses through RecE/T-mediated LLHR into plasmid vectors has not been demonstrated. Reports about cloning of influenza A virus genome segments by recombination in RecA expressing *E. coli* DH5α target the same aim of efficient recombinatorial cloning^32^,^33^, but only resulted in 5–10 ampicillin resistant clones per reaction, showing that the system was less efficient and not usable for higher throughput applications. By using a RecE/T system in which full-length RecE has been used to mediate LLHR in the absence of plasmid replication^27^, we describe here a highly efficient and controllable system. With selection of 200 to 1000 resistant colonies after recombination of RABV full length cDNAs and insertion rates up to 88% (Fig. 4B), we conclude that the methodology can not only serve as a tool to rapidly and cost-effectively generate viral full length cDNA clones for mutagenesis and recovery of recombinant viruses but also is highly beneficial for high-throughput sequence analyses at a clonal full length cDNA genome level. Combination with single clone analysis by sequencing of an adequate number of full length cDNA clones allows identification of combined SNPs in cases such as RABV, where sequence variations are low and few SNPs are distributed all over the complete genome. This can also be achieve with state of the arte long-read sequencing technologies, but it has to be stated that Oxford Nanopore sequencing with error rates reported in the range of 5–40% currently does not reach sufficient read quality. Pacific Biosciences SMRTbell technology with single read accuracy of up to 100% of enables reliable single molecule sequencing. Nevertheless, like sequencing individual plaques, it does not aid in generating clones of the virus unless the genome is synthesized according to the generated sequence.

One further example for the power of the system is given by the combined selection of two amino acid exchanges (D266N and K508T) in the glycoprotein G after one passage of a fox derived RABV isolate in a heterologous raccoon host (Table 5). By comparison to a deep sequence of the progenitor fox isolate it was also possible to show that both D and N residues already existed in the authentic virus population at frequencies of nearly 50% (Table 2), whereas the K508T detected in all 4 clones may have been acquired during the raccoon passage. Although these data do not yet provide evidence for the selection of raccoon-adaptive mutations without sequencing of further full-length clones for statistical analysis, with the successful rescue of D266N and K508T mutant recombinant rRABVfox(rac) viruses from clone 3 and separation from other mutant sequences (Table 5) this example demonstrates the potential of the system for further approaches targeting sequence variability and adaptive processes in virus infections. Interestingly, it was speculated that higher doses of fox RABV are required to provoke disease in raccoons^30^,^34^. Whereas raccoons and foxes are both carnivores and only minor adaptation to new hosts may be needed for cross species transmission of RABV, sustained spill overs to less related host species may require further adaptive mutations.

Notably, by comparing RABVs circulating in Namibian kudu and jackal population amino acid replacements in the glycoprotein of Kudu-derived RABVs have been predicted to cause adaptation to Kudu-hosts^35^, but an experimental basis for downstream verification of the adaptive character of the respective mutants in the specific genetic background was lacking. The strategy described here would allow the rapid cloning, mutagenesis and recovery of virus mutants from sustained spillovers to allow experimental proof of genetic adaptation of RABVs to new hosts.

With a maximum of 5 single nucleotide exchanges in 8 completely sequenced full length clones, the frequency of detected mutations was low. Although we cannot exclude introduction of mutations during reverse transcription and PCR amplification of full length cDNAs, pre-existence of silent and non-silent mutations in the progenitor fox and dog sequence populations indicates that at least part of the identified mutations represent minor variations within the virus population and were not generated during the cloning process.

Because of the low degree of sequence variations only four clonal sequences were required to identify cDNA clones that were identical to the consensus sequence of the progenitor virus isolates (Table 3). In case of the dog RABV derived cDNA clones (rRABV-Dog), two of the four clones exhibited 100% identity at the nucleotide level. Thus, even at a risk of error prone RT-PCR amplification a limited number of clones is sufficient for the generation of wild-type virus like clones, indicating that the error rate was low enough to generate wt RABV virus clones.

Accordingly, recombinant wild-type virus like rRABV-Fox clone 9 and rRABV-Dog clone 14 exhibited growth kinetics comparable to their parental virus isolates (Fig. 5), even though one amino acid exchange was detected in the N gene of rRABV-Fox clone 9 (Table 3). The dog and fox virus specific degree of growth attenuation on non-neuronal BSR T7/5 cells further confirmed the wt RABV like virus growth, strongly supporting that the here described technology led to the rapid generation of recombinant wt RABV like viruses.

It is well known that lyssavirus field isolates are superior to lab strains in infecting mice from the periphery. For instance, lab strain challenge virus standard 11 (CVS-11) virus is only partially lethal after intramuscular inoculation of 5 × 10^5^ TCID_50_/mouse, whereas comparable infections with non-fixed isolates such as European Bat Lyssaviruses (EBLVs) or Bokeloh Bat Lyssavirus (BBLV) are 100% lethal in 3 week old BALB/c mouse at those doses^36^. Here, comparable and progenitor virus specific outcomes of i.c. inoculations (Fig. 6A,C) and 100% lethality after i.m. inoculation of both recombinant viruses at lower doses of 5 × 10^3^ TCID_50_/mouse strongly supported the wt virus character of those clones. Notably, a higher lethality of rRABV-Dog 14 when compared to the parental wt RABV Dog showed, that use of a clonal virus can be superior in pathogenesis, maybe by the absence of attenuating virus variants. In view of the low level of sequence variability in both, the original dog and fox virus isolates (Table 2), further analysis of multiple clones may reveal whether the overall outcome of RABV infection indeed is affected by the limited number of sequence variants.

From growth kinetics and peripheral mouse inoculations we conclude that the described blueprint for cloning and generation of recombinant wild-type RABV indeed allows the recovery of non-fixed recombinant viruses for further analyses related to RABV pathogenesis in relevant hosts. This possibility is new since so far only a limited number of recombinant RABVs^12^,^22^ is available derived from viruses which had not been adapted to cell culture or mouse models. As now a reliable method to generate such viruses is available, one of the future steps will be generation of suitable cell culture systems from relevant virus hosts for virus rescue and amplification.

Although it has been shown that additional genes can be inserted in recombinant RABVs and other non-segmented negative strand RNA viruses without effects on the virus replication^37^, in view of the low cell culture replication level of non-fixed viruses we asked whether rescue and subsequent replication of recombinant non-fixed RABV is affected by the insertion of an additional reporter gene encoding transcription unit. Expression of fluorescent reporters from such viruses would greatly enhance the potential to study the tropism and pathogenicity within natural host species. Successful recovery of boht rRABV-Dog Katushka and rRABV-Fox Katushka expressing red fluorescent protein showed that integration of an additional transcription unit into the non-fixed genetic background did not heavily interfere with virus rescue. However, about 1 log decreased virus titers of the reporter expressing viruses in growth curves (Fig. 5) indicated that virus replication was affected to some extend, whereas cell specific replication measured by the drop in virus titres on BSR T7/5 cells was still wt RABV like. Further pathogenesis studies have to await to clarify, whether the detected drop in virus replication affects virus pathogenicity *in vivo* and whether such reporter viruses are usable tools to investigate RABV pathogenesis by tracking reporter expressing virulent and non-fixed RABV *in vivo*.

The virulence of the parental dog and fox RABVs had been demonstrated previously in experimentally infected raccoons^30^: high lethality within 8 to 11 and 12 to 20 days post infection with dog and fox RABVs, respec**t**ively, together with limited cell culture replication shown here (Fig. 5) indicate that we indeed cloned and rescued virulent, non-fixed RABVs of dog and fox, although virulence for the recombinant viruses remains to be determined in the carnivore model.

The flexibility of the system also allows easy and quick adaptation to other viruses. Cloning of an orthobunyavirus L segment RNA after determination of the genome termini through RNA-ligation at an efficiency of 100% and concurrent modification of the plasmid vector by the used PCR primers to generate a BATV specific hammerhead ribozyme sequence comprising a reversed copy the first 9 nts of the BATV genome end shows that the system is applicable to other, unrelated negative strand RNA viruses.

Overall, RecE/T cloning of full length cDNA copies from negative strand RNA viruses is a fast and efficient method to generate recombinant viruses and to make virus sequence populations accessible to sequence analysis at a clonal level. We therefore believe that the system will substantially contribute to future investigations of negative strand RNA virus biology. Moreover, as it may not be restricted to negative strand RNA viruses, it may also allow rapid phenotypic characterization of clinically relevant virus isolates and e.g. drug resistance testing. The now available recombinant RABVs from virulent and non-fixed parental street viruses together with the possibility of targeted mutagenesis and reporter gene insertion may greatly enhance the possibilities in reservoir orientated pathogenesis RABV research.

## Methods

### Cell culture and viruses

BSR T7/5 cells were cultivated as described previously^11^. Murine neuroblastoma cells (Na42/13) were used for virus amplification. All cells were provided by the Collection of Cell Lines in Veterinary Medicine (CCLV), Friedrich-Loeffler-Institut Riems, Germany.

Recombinant virus SAD L16 was derived from vaccine strain SAD B19^1^. Street RABV isolates from dog (Azerbaijan 2002) and fox (Germany 1998) have been described previously^30^. RABV-Fox(raccoon) was re-isolated after one experimental passage in raccoon^30^. Batai Virus isolated in 2009 in Southwest Germany^31^ was the donor for L segment RNA.

### Vector construction and amplification of minimal recombination vector sequences

Plasmid pCMV HaHd miniGFP comprised a GFP coding RABV minigenome sequence. The minigenome sequence was upstream flanked by cytomegalovirus immediate early and bacteriophage T7 RNA-Polymerase promotors and a synthetic, RABV-specific hammerhead ribozyme sequence. Downstream, a hepatitis delta ribozyme sequence was located^12^. To reduce the vector size, DNA fragments were amplified from pCMV HaHd miniGFP with the primers miniP CMV/miniP-*EcoR*I-rev and miniP-*Mfe*I-for/miniP-*Nhe*I-rev (for primer sequences see Supplementary Table S3). After restriction enzyme digest the fragments were ligated to pHaHdmin (see Supplementary Fig. S1 for plasmid organization).

The recombination vector pHaHdmin was linearized with *Bgl*II and used as a template for PCR amplification of a linear 2.7 kb vector fragment. By adding 50 nucleotide-long virus specific homology arms to the primers (see Supplementary Table S3), the terminal RABV sequences of the leader and trailer regions were attached to the PCR products. To remove residual pHaHdmin plasmid, PCR samples were digested with *Dpn*I for 1 h at 37 °C (5U per PCR reaction in PCR buffer) to destroy methylated plasmid DNA. The PCR-products were then gel purified (QIAquick Gel Extraction Kit, Qiagen) and subjected to LLHR.

### RNA Isolation

Virus RNA was extracted from supernatant virions after polyethylenglycol (PEG) precipitation (PEG Virus Precipitation Kit; Abcam) or from infected BsrT7/5 or Na42/13 cell cultures with PeqGOLD TriFast™ reagent (Peqlab) according to the supplier’s recommendations.

### RNA ligation and RT-PCR sequencing

To determine the terminal sequences of RABV genomes, the viral RNA genomes isolated from PEG precipitated supernatant virions were circularized by T4 RNA Ligase. To remove the 5′-triphosphate residues from genomic RNAs prior to RNA ligation, 1 μg RNA was digested with 5 units of 5′ Pyrophosphohydrolase (RppH; New Englang Biolabs) in 20 μl reaction buffer (50 mM NaCl, 10 mM Tris-HCl, 10 mM MgCl_2,_ 1 mM DTT, pH 7.9) and was then purified (RNeasy mini kit, QIAGEN) according to the manufacturer’s recommendations. 400 ng of RppH digested or not digested genomic RNA were ligated with 10 units T4 RNA Ligase (New England Biolabs) in 20 μl reaction buffer (50 mM Tris-HCl, 10 mM MgCl_2_, 1 mM DTT, 1 mM ATP, pH 7.5) for 1 h at 37 °C. The reaction was stopped by adding EDTA to a final concentration of 5 mM and 15 min incubation at 65 °C. Five μl of the ligation sample was directly used as a template for RT-PCR with SuperscriptIII reverse transcriptase (Invitrogen) and GoTaqFlexi (Peqlab) according to the manufacturer’s protocols with gene specific oligonucleotides listed in Supplementary Table S3. PCR products were gel purified (QIAquick Gel Extraction Kit, QIAGEN) and sequenced on an ABI genetic analyzer (Applied Biosystems) using the BigDye Terminator v3.1 Cycle Sequencing Kit (LifeTechnologies) with the same oligonucleotides used for PCR amplification (see Supplementary Table S3).

### Long range RT-PCR of virus genomes

For amplification of full genome cDNA copies of different RABVs specific primer pairs (see Supplementary Table S3) were used. The B19-for/B19-rev primer pair was used for amplification of a full genome cDNA copy of the SAD L16 virus. The primer pair RABV-for/RABV-rev was used to amplify full length copies of dog and fox derived RABV street isolates. The primer pair BATV-Lseg/Lseg-rev was used for amplification of a Batai virus L segment cDNA. RT-PCR amplification of the 11.9 kb RABV full genome and the 6.9 kb BATV L segment cDNA was performed with 400 units SuperscriptIII reverse transcriptase (Invitrogen) for 1 h at 55 °C and high fidelity Phusion Polymerase (Promega). Individual PCR reaction contained 0.25 μl cDNA from 1 μg reverse transcribed vRNA, 0.2 μM forward and reverse Primer, 0.2 mM dNTPs and 0.5 units Phusion polymerase in 1 × HF reaction buffer. Resulting PCR products were gel purified (QIAquick Gel Extraction Kit, QIAGEN) and were then used for LLHR.

### Linear-to-linear RedE/T recombination in *E. coli*

Recombination of linear long range RT-PCR products and linear plasmid vector derived PCR products was performed by electroporation of both DNAs in the *E. coli* strain GB05-dir (Genebridges). Briefly, 1.4 ml Luria Broth medium was inoculated with 30 μl of an overnight GB05-dir culture. After reaching an OD600 of 0.3, the RecE/RecT recombinases were induced by adding L-Arabinose to a final concentration of 0.30–0.35% (w/v). A not induced culture was used as a negative control. 35 min after induction, the bacteria were pelleted at 11.000 rpm and 2 °C for 30 and washed three times with ice-cold water, supernatants were discarded. 100 ng of gel-purified linear minimal vector and 100 ng of genomic virus cDNA in a maximal volume of 15 μl H_2_O were added to the pelleted bacteria. Electroporation was performed in a 0.1 mm gapped cuvette at 1.35 kV (MicroPulser™, Biorad). Subsequently, the bacteria were resuspended in 1 ml LB-medium and were incubated for 90 min at 37 °C. Recombination events were selected by over-night cultivation of the bacteria on LB Agar plates containing 100 μg/ml ampicillin. Ampicillin resistant colonies from induced and not-induced approaches were compared to estimate the efficiency of the recombination process. Insertion of cDNA genome copies into the vector was checked after plasmid preparation from individual clones by *Sac*I digest.

### Virus rescue

Recombinant RABVs (rRABVs) were rescued by co-transfection of full length genome cDNA and expression plasmids into BSR T7/5 cells as described previously^11^,^29^. For all RABV rescues the expression plasmids pCAGGS-N, pCAGGS-P and pCAGGS-L encoding the N, P and L gene sequences of attenuated SAD B19^38^ under the control of an RNA polymerase II promoter have been used. After 3 to 6 days, supernatants of transfected cultures were transferred to Na42/13 neuroblastoma cells. Two days after transfer, positive virus rescues were identified by indirect immunofluorescence with RABV N- and G-specific antibodies. Recombinant viruses were further amplified by standard methods on Na42/13 cells. Infectious virus titers in cell culture supernatants were determined by end point dilution and titration on Na42/13 and BSR T7/5 cells.

### Next generation sequencing (NGS)

For sequencing of the parental RABV isolates from fox (Germany 1998) and dog (Azerbaijan, 2002), total RNA was extracted from cell culture supernatant using peqGOLD TriFast (Peqlab, VWR International GmbH) and RNeasy mini kit (Qiagen) as described^39^. Double stranded cDNA was generated using a cDNA synthesis system kit (Roche) and random hexamer primers. For library preparation of full length genome cDNA clones and parental isolates, a portion of 1 μg double stranded cDNA was fragmented (Covaris M220; Covaris) and transformed to barcoded sequencing libraries using GeneRead DNA Library L Core Kit (Qiagen) according to the manufacturer’s instructions The resulting libraries were, size-selected using AMPureXP beads (Beckmann-Coulter), qualified (Bioanalyzer 2100; Agilent) and quantified with the Ion Library Quantitation Kit (Life Technologies) and thereafter prepared for equimolar multiplexed sequencing using the Ion 316 Chip Kit v2 on the Ion PGM System (Life Technologies) according to the manufacturer’s instructions. Ion PGM Hi-Q chemistry (Life Technologies) was used for template preparation and sequencing. The resulting raw reads were assembled using the 454 Sequencing Systems Software (v2.6; Roche) and were thereafter mapped along their respective consensus sequence for variant and SNP calling, using the same software (v3.0; Roche). Whole genome sequences of dog (LN879480) and fox (LN879481) isolate were deposited in the INSDC sequence database. Variant frequencies for dog and fox were calculated from read counts per position where sufficient sequence depth (>150X) was available. Only variants present on both forward and reverse reads and with an abundancy of ≥5% were taken into account, hence only variants with a confidence level of ≥99.999% according to^28^ were considered.

### Indirect Immunofluorescence

Indirect immunofluorescence with mouse monoclonal antibody E559^40^ or rabbit polyclonal antibody N-161^12^ recognizing RABV G protein or RABV N protein, respectively, and AlexaFluor 488 conjugated secondary antibodies (Molecular Probes) was performed by standard techniques after fixation with 3.0% paraformaldehyde in PBS and permeabilization with 0.5% Triton-X100 in PBS. Images were acquired with a Leica SP5 confocal laser scan microscope (63 × objective; numerical aperture: 1.4) with sequential acquisition of the fluorophores in double fluorescent specimen. Images were processed with the ImageJ software version 1.48b^41^.

### Insertion of red fluorescent marker into pRABV-Dog 14 and pRABV-Fox 9

For insertion of a red fluorescent protein marker into recombinant pRABV-Dog 14, the N/P gene border sequence of SAD L16 comprising the conserved RABV transcription stop/start signal sequences was integrated by PCR amplification of 0.28 and 0.55 kb DNA-fragments with the primers Dog-KpnI-for/Dog-MCS-rev and Dog-MCS-for/Dog-*EcoR*I-rev, *Sac*II digestion of both fragments, ligation and second round PCR amplification of a 0.82 kb DNA-fragment with the primers Dog-*Kpn*I-for/Dog-*EcoR*I-rev. A 0.80 kb *Kpn*I/*EcoR*I fragment from the product was inserted into *Kpn*I/*EcoR*I digested pRABV-Dog. The resultant pRABV-Dog MCS was *Nhe*I/*Not*I digested and ligated with a 0.74 kb *Nhe*I/*Not*I fragment digested from pSADL16 Katushka (unpublished) containing the ORF of the red fluorescent marker. pRABV-Fox Katushka was constructed by a similar strategy. After PCR amplification of DNA fragments from pRABV-Fox clone 9 with primer pairs Dog-KpnI-for/Dog-EcoRI-rev and Dog-MCS-for/ Fox-Eco72I-rev (Supplementary Table S3) the products were concatenated through SacII and the ligation product was further amplified with the primer pair Dog-KpnI-for/Fox-Eco72I-rev, digested with KpnI and Eco72I and inserted into KpnI/Eco72I digested pRABV-Fox clone 9.

### Animal experiments

The animal experiments were evaluated by the responsible animal care, use and ethics committee the State Office for Agriculture, Food Safety and Fishery in Mecklenburg-Western Pomerania (LALFF M-V) and gained governmental approval (registration number LALLF M-VTSD7221.3-2.1-00211). General care and methods used in the animal experiments were carried out according to the approved guidelines. The animals were checked daily for clinical signs and were euthanized after development of severe clinical signs of rabies indicated by (trembling, spasms, weight loss to more than 20%, paralyses, coma). Three to four week old BALB/c mice were inoculated by the intracranial (i.c.) route of infection with 30 μl virus suspension at a dose of 50 focus forming units (ffu) per mouse (6 mice per group). Intramuscular (i.m.) inoculations were performed by injection of 30 μl of virus suspension (50 ffu/mouse or 5000 ffu/mouse) in the thigh of one hind leg. All inoculations were performed under anaesthesia (Isofluran).

## Additional Information

**How to cite this article**: Nolden, T. *et al.* Reverse genetics in high throughput: rapid generation of complete negative strand RNA virus cDNA clones and recombinant viruses thereof. *Sci. Rep.* **6**, 23887; doi: 10.1038/srep23887 (2016).

## Supplementary Material

Supplementary Information

## Figures and Tables

**Figure 1:**
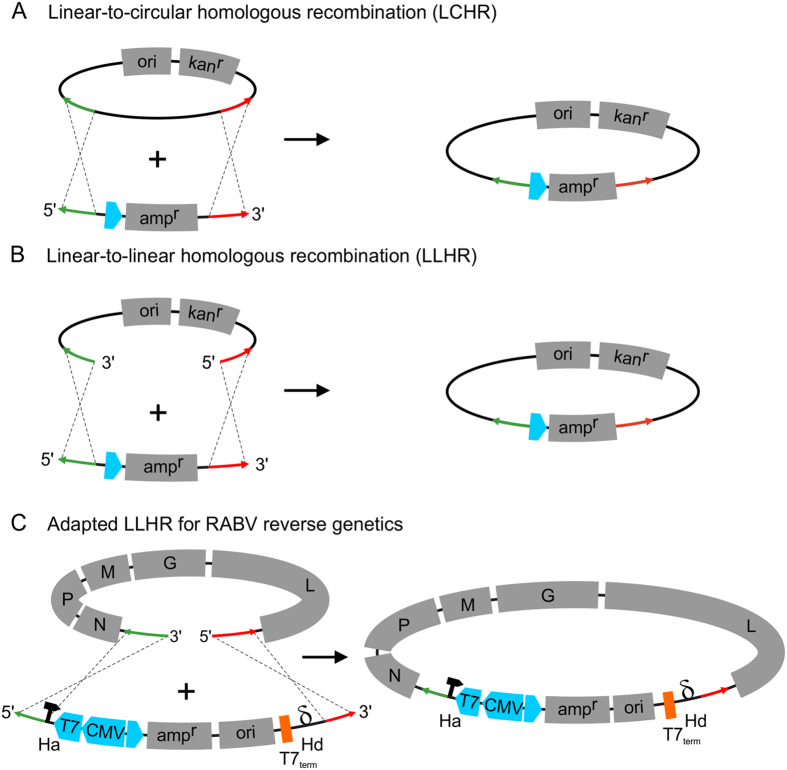
Comparison of Linear-to-circular (LCHR) with Linear-to-linear (LLHR) homologous recombination and adapted LLHR for RABV reverse genetics. While LCHR (**A**) depends on active replication, LLHR (**B**) uses a different mechanism and can be adapted to RABV reverse genetics vector cloning (**C**). The linear vector codes for an ampicillin resistance gene (ampR) and contains an origin of replication for plasmid propagation. Additionally, the minimal linear vector contains T7 and CMV promotors (in blue) and a T7 termination signal (in orange) for transcription termination and polyadenylation. Ribozyme sequences flank the RABV virus cDNA after LLHR recombination (Hammerhead ribozyme, Ha; Hepatitis δ ribozyme, Hd). Homologous sequences required for recombination between target molecules and vector sequences are specified by red and green arrows.

**Table 1:** Oligonucleotides for PCR amplification of the linear minimal vector from pHaHdmin.

Id	sequence
L16 Tr	**TTTGGTTGTTTGATTGTTTTTCTCATTTTTGTTGTTTATTTGTTAAGCGT**GGGTCGGCATGGCATCTCCAC
L16 Le	**GCTTTGCAATTGACAATGTCTGTTTTTTCTTTGATCTGGTTGTTAAGCGT**GACCCGGGACTCCGGGTTTCGTC
Dog Tr	**TcTGGcTGcTTGATTGTTTTTtcCATcTTTaTTGTTTtTTTGTTAAGCGT**GGGTCGGCATGGCATCTCCAC
Dog Le	**GCTTTGCAAcTGACgcTGTCTGcTTcTTCTcTGATtTtGTTGTTAAGCGT**GACCCGGGACTCCGGGTTTCGTC
Fox Tr	**TcTGGcTGTTTGATTGTTTTTtcCATcTTTaTTGTTTtTTTGTTAAGCGT**GGGTCGGCATGGCATCTCCAC
Fox Le	**GCTTTGtAAcTGACgcTGTCTGcTTcTTCTcTGATtTtGTTGTTAAGCGT**GACCCGGGACTCCGGGTTTCGTC

Bold: virus isolate specific terminal leader (Le) and trailer (Tr) coding sequences used as “homology arms” for linear to linear recombination. Single nucleotide polymorphisms referring to SAD L16 are indicated by lower case letters. Non-bold letters: vector specific sequences.

**Table 2:** Nucleotide Variations in wt RABV-Fox and wt RABV-Dog isolates.

A: Nucleotide Variations in wt RABV-Fox isolate							
nt position	A	G	C	T	silent	non-silent	in rRABV
4015	0.00%	0.33%	76.24%	23.43%	yes	–	fox 9, 10
4111	51.99%	48.01%	0.00%	0.00%	–	N266D [G]	fox 8, 9, 10, 11
4655	0.00%	0.00%	94.35%	5.65%	–	S466L [G]	–
11294	51.92%	48.08%	0.00%	0.00%	yes	–	–
**B: Nucleotide Variations in wt RABV-Dog isolate**							
2469	12.17%	87.61%	0.00%	0.00%	yes	–	–
2501	5.42%	0.00%	94.58%	0.00%	–	N2K [M]	–
4243	94.50%	0.18%	5.32%	0.00%	–	M310L [G]	–

The variation of nucleotides beyond the level of consensus sequences was determined in the datasets of the isolates RABV-fox (average depth 797X) and RABV-dog (average depth 405X). Only variants at least fulfilling the minimum requirements of 28 for the detection of variants in homopolymeric regions with a confidence level of at least 99.999% were taken into account. Four non-silent mutations the affected amino acid positions and ORFs (brackets) are indicated. Clones with identified sequence variations are listed.

**Table 3:** Sequences of individual RABV-Fox and RABV-Dog cDNA clones in comparison to the consensus sequence of the parental RABV-Fox and RABV-Dog virus populations.

A: cDNA clones pRABV-Fox												
	nucleotide exchanges	amino acid exchanges										
	11923 nt	–	N	–	P	–	M	–	G	–	L	–
fox8	3		M60T		–		–		–		–	
fox9	4		V23A		–		–		–		–	
fox10	3				–		–		–		–	
fox11	5				–		P45S		–		I150V, V1183I, E1522Q	
**B: cDNA clones pRABV-Dog**												
dog3	4		–		–		–		F14S, L451P		A408V	
dog5	0		–		–		–		–		–	
dog14	0		–		–		–		–		–	
dog15	1		–		–		–		–		–	

The numbers of nucleotide changes within the 11923nt virus genomes are indicated. For non-synonymous single nucleotide exchanges (SNP) in the N, P, M, G or L protein sequence the position in the virus protein and the resulting amino acid exchange are provided.

**Table 4:** Rescue efficiencies of RABV-Fox (A) and RABV–Dog (B) cDNA clones in BSR-T7/5 and virus replication after supernatant passage to (Na42/13) neuroblastoma cells.

A: Rescue efficiencies rRABV-Fox			B: Rescue efficiencies rRABV-Dog		
rRABV-Fox	Recovery onBSR-T7/5	Replication afterpassage to Na42/13	rRABV-Dog	Recovery onBSR-T7/5	Replication afterpassage to Na42/13
fox8	0/3	no	dog3	1/3	no
fox9	3/3	yes	dog5	3/3	yes
fox10	3/3	yes	dog14	3/3	yes
fox11	2/3	no	dog15	3/3	yes

Recues in BsrT7/5 cells were performed in triplicates. The rescue efficiencies after transfection into BSR T7/5 cells (number of virus positive approaches/number of trials) are provided. Successful virus passage to neuroblastoma cells and autonomous virus replication is indicated in the last column.

**Table 5:** Sequences of individual RABV-Fox cDNA clones after experimental infection of a raccoon (rac) in comparison to the consensus sequence of the parental RABV-Fox population.

cDNA clones pRABV-Fox(rac)													
	Nucleotide exchanges	Amino acid exchanges											Rescue
	11923 nt	–	N	–	P	–	M	–	G	–	L	–	
fox(rac)1	5		–		–		–		D266N*, K458T, K508T		–		yes
fox(rac)2	5		–		–		–		D266N*, K508T		V328I		no
fox(rac)3	3		–		–		–		D266N*, K508T		–		yes
fox(rac)4	5		–		–		–		D266N*, K508T		T118A, W925R		no

The numbers of nucleotide changes within the 11923nt virus genomes are indicated. For non-synonymous single nucleotide exchanges (SNP) in the N, P, M, G or L protein sequence the position in the virus protein and the resulting amino acid exchange are provided. *Non-synonymous SNP that was already preformed in the parental RABV-Fox population. Successful rescue of recombinant viruses from the clones is indicated in the last column.
