# Supplementary material for: Reverse genetics in high throughput: rapid generation of complete negative strand RNA virus cDNA clones and recombinant viruses thereof
Source: Sci Rep. 2016 Apr 5;6:23887. doi: 10.1038/srep23887 (PMC4820695; doi:10.1038/srep23887)
Supplement: Supplementary Information [file srep23887-s1.pdf]

Reverse genetics in high throughput: rapid generation of complete negative strand RNA virus cDNA clones and recombinant viruses thereof.

[1]NOLDEN T, [2]PFAFF F, [1]NEMITZ S, [1]FREULING CF, [2]HOEPER D, [1]MUELLER T AND [1*]STEFAN FINKE

[1]Friedrich-Loeffler-Institut, Federal Research Institute for Animal Health, Institute of Molecular Virology and Cell Biology, D-17493 Greifswald – Insel Riems, Germany

[2]Friedrich-Loeffler-Institut, Federal Research Institute for Animal Health, Institute of Diagnostic Virology, D-17493 Greifswald – Insel Riems, Germany

Supplementary Figure S1

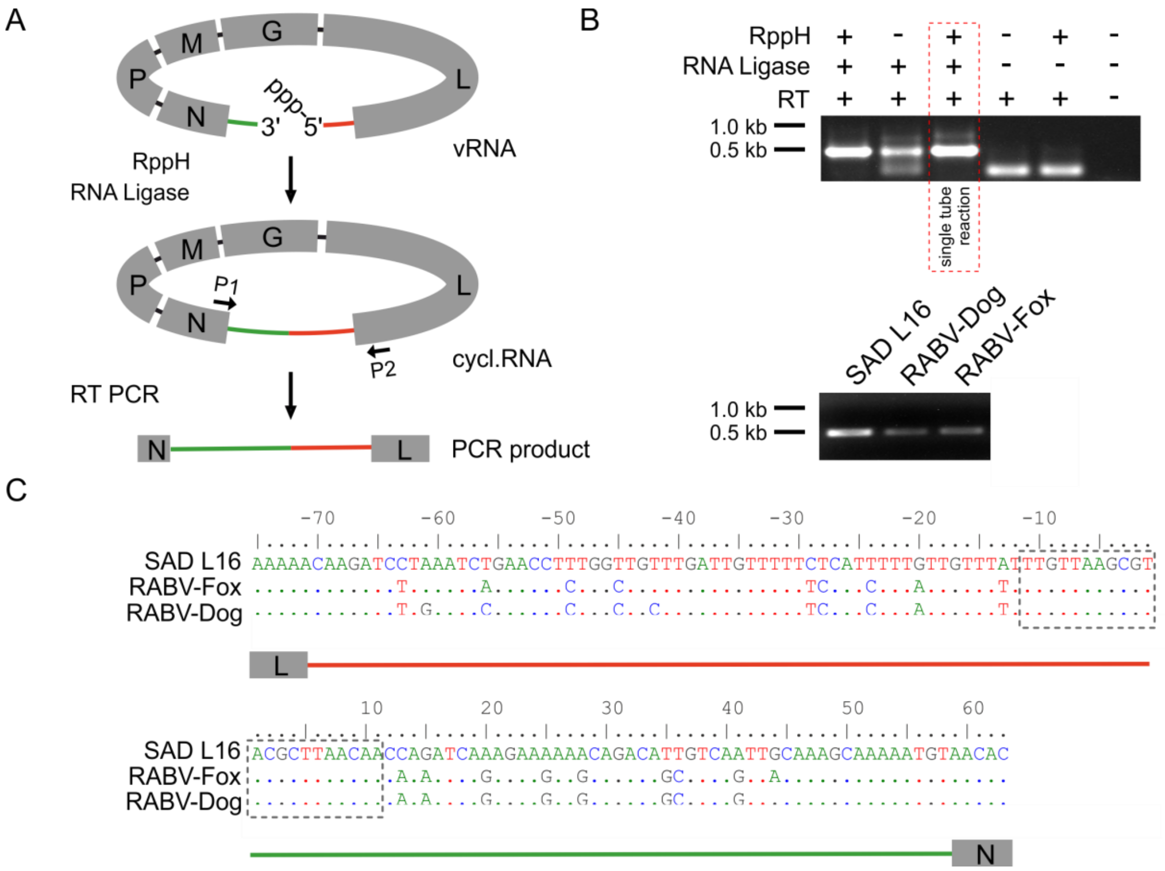

Determination of virus leader and trailer sequences by RNA T4 ligase-mediated endjoining

(A) Schematic presentation of T4-RNA mediated RNA end-joining for determination of virus end-sequences. The oligonucleotide L16endseq (P2) was used for priming the reverse transcriptase reaction before amplification and sequencing of the PCR product spanning the RNA ligation site. (B) RT-PCR products of T4-RNA ligase-mediated end-joining. Positive RT-PCR signals were obtained, when RppH digestion was performed as separate reaction or as in a single tube reaction together with T4-RNA ligase (lane1 and lane3, respectively). Without removing 5'triphoshate, RNA end-joining was less effective (lane 2). No PCR products were obtained when T4 RNA ligase or reverse transcriptase was absent (lane 4, 5 and lane 6, respectively). (C) Sequence alignment of SAD L16, RABV-Dog and RABV-Fox end-sequences. For reference SAD B19 leader and trailer sequences were concatenated.

Supplementary Figure S2

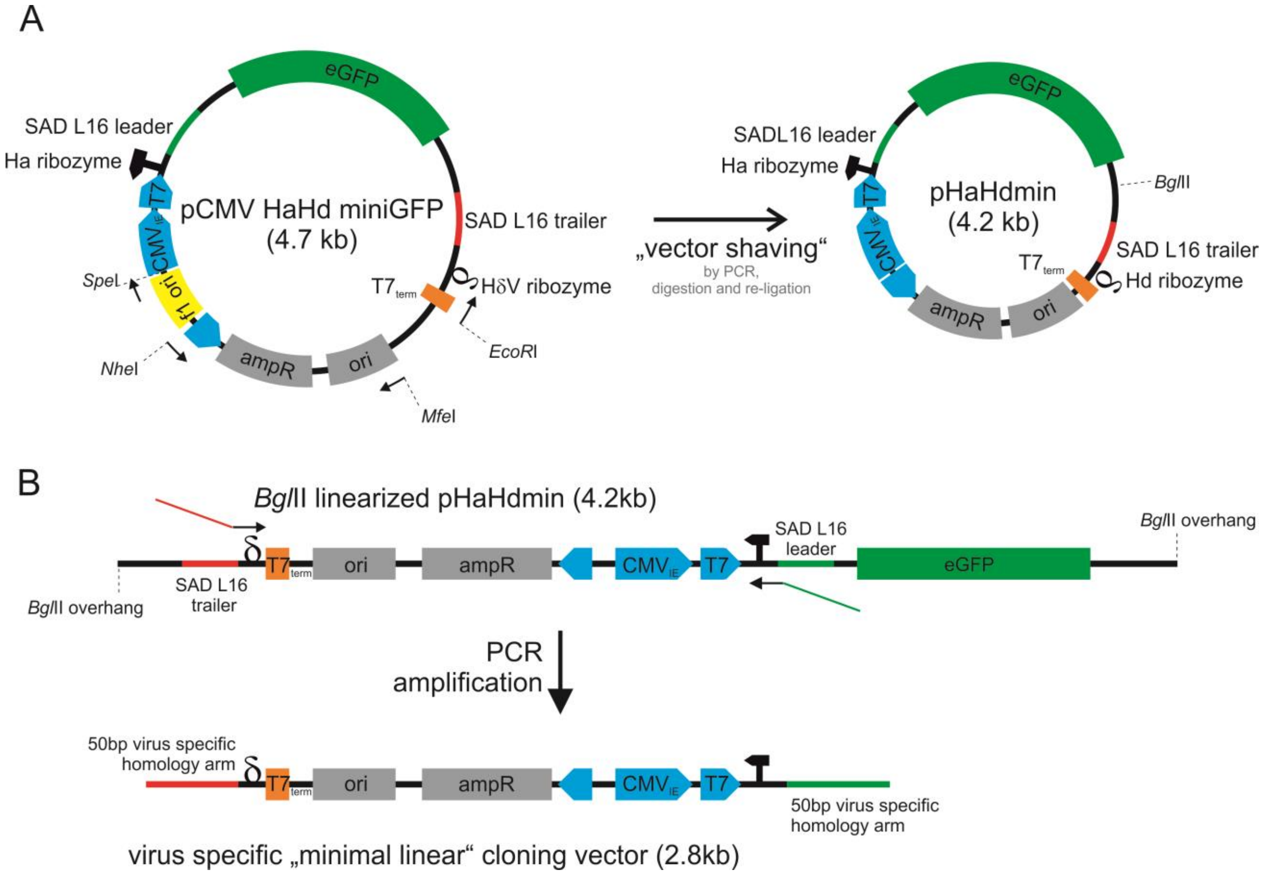

Construction of pHaHdmin and PCR amplification of a linear minimal cloning vector.

(A) pHaHdmin was generated by amplification of two PCR fragments from pCMV HaHd miniGFP and re-ligation via *Spe*I / *Nhe*I and *Mfe*I / *EcoR*I compatible ends introduced by the 5′ ends of the PCR oligonucleotides (indicated by arrows) . The "vector shaving" decreases the size of the minimal linear cloning vector amplified from pHaHdmin by PCR and reduces the possibility of intramolecular recombination in subsequent steps of RecE/T recombineering. The 50bp homology arms, compatible to RABV leader (green) and trailer (red) sequences were introduced by the pHaHdmin-specific oligonucleotides which binds within the ribozyme sequences of the vector pHaHdmin (B). Promotor sequences, that drives virus cDNA and ampR gene transcription are colored blue; CMV_{IE}, Cytomegalievirus imidiate early promotor; T7 and T7 $_{term}$, promotor and terminator signal sequences of T7 bacteriophage; ori, origin of replication.

Supplementary Figure S3

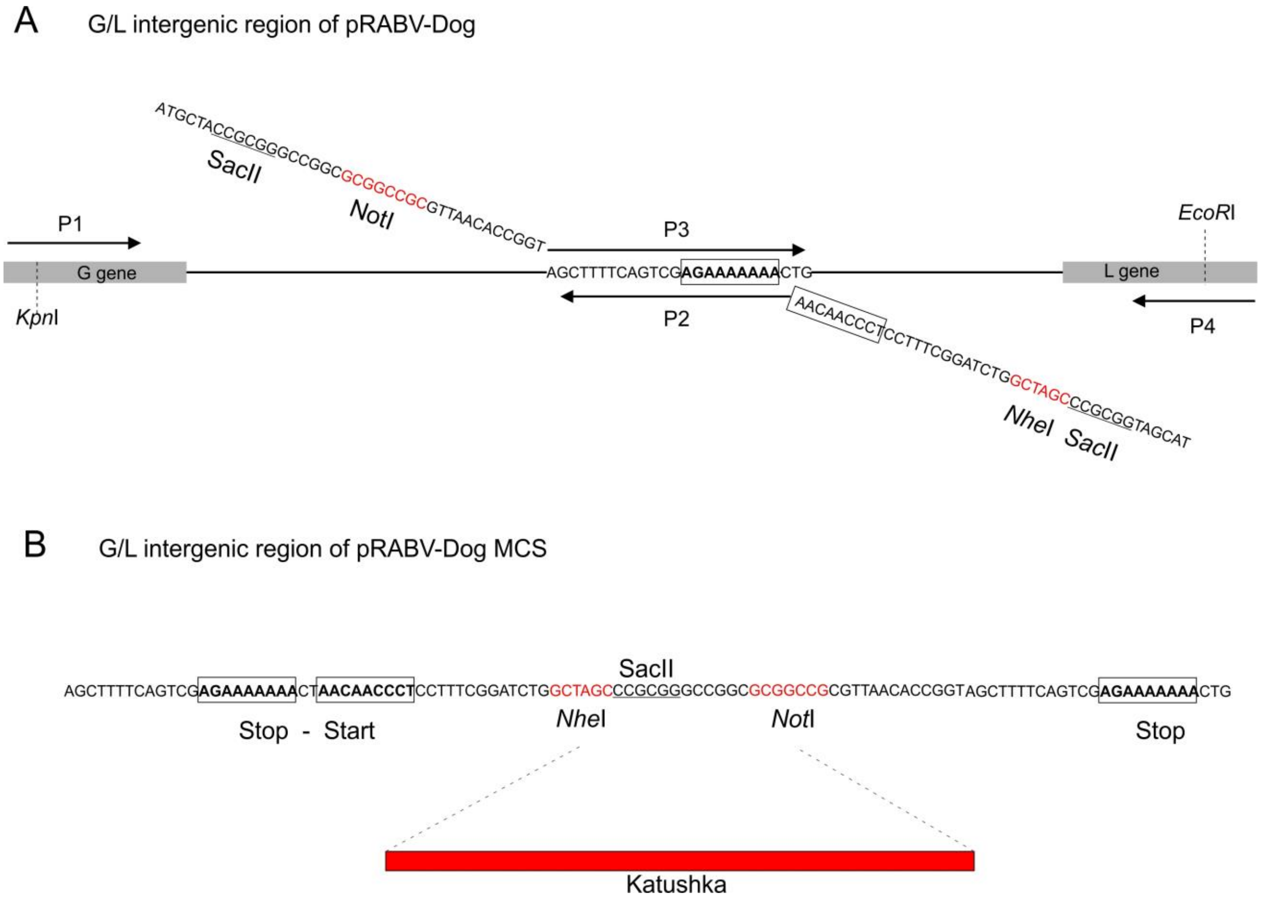

Insertion of the MCS with an additional RABV Start-Stop signal and the fluorescent marker Katushka into pRABV-Dog and pRABV-Fox. The G/L intergenic region of pRABV-Dog (A) was amplified with primer pairs P1/P2 and P3/P4 (see Supplementary Table S3). Generated PCR products were concatenated using the *Sac*II site within the primer 5′ overhangs (underlined). The resulting product with an additional stop-start signal (framed) was re-amplified with P1 and P4 and subsequently cloned into pRABV-Dog via *Kpn*I/*EcoR*I to yield pRABV-Dog MCS (B). The fluorescent marker Katushka from pSAD L16 Katushka (unpublished) was inserted in a second step into pRABV-Dog MCS via *Nhe*I and *Not*I sites (in red).
pRABV-Fox Katushka was constructed in a similar strategy was used (see material and methods section).

Supplementary Figure S4

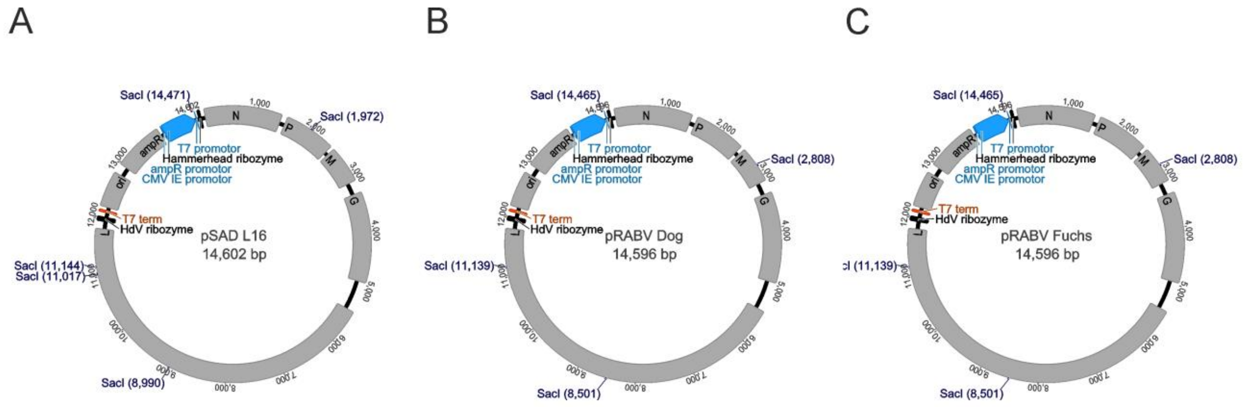

	Fragment start	Fragment end	Fragment length
pSAD L16	1972	8989	7018
	8990	11016	2027
	11017	11143	127
	11144	14470	3327
	14471	1971	2103

	Fragment start	Fragment end	Fragment length
pRABV Dog	2808	8500	5693
	8501	11138	2638
	11139	14464	3326
	14465	2807	2939

	Fragment start	Fragment end	Fragment length
pRABV Fox	2808	8500	5693
	8501	11138	2638
	11139	14464	3326
	14465	2807	2939

Supplementary Table S4

pCMVHAHd GFPmini "vector Shaving"

primer	sequence	comment
miniP-CMV-for	CGTTGACATTGATTATTGAC	
miniP-EcoRI-R	ATCGTGGAATTCATATAGTTCCTCCTTTCAGC	
miniP-MfeI-F	ATCGTGCAATTGAGGCCAGCAAAAGGCCAGGA	restriction sites, underli
miniP-NheI-R	ATCGTGGCTAGCAAATACATTCAAATATGTAT	

RABV long range PCR

primer	sequence	comment
B19-for	tcgatcccgggtcacgcttaacaaccagatca	
B19-rev	taatacacctgcccatgccgacccacgcttaacaaataaacaa	leader/trailer, red
RABV-for	tcgatcccgggtcACGCTTAACAACAAAATCA	
RABV-rev	taatacacctgcccatgccgacccACGCTTAACAAAAAAACAA	

Katushka cloning

primer	sequence	comment
Dog-MCS-for (P3)	ATGCTACCGCGGGCCGGCGCGGCCGCGTTAACACCGGTAGCTTTTCAGTCGAGAAAAAAACTG	SacI underlined, MCS in r
Dog-MCS-rev (P2)	ATGCTACCGCGGGCTAGCCAGATCCGAAAGGAGGGTTGTTAGTTTTTTTCTCGACTGAAAAGC	
Dog-KpnI-for (P1)	TCCAGGAACTGGTACCAAAGG	
Dog-EcoRI-rev (P4)	CCTCGGGGGGAATTCTCAACCCTC	
Fox-Eco72I-rev (P5)	GAGTTTCCACACGTGGACAAG	

Batai Virus specific endsequencing and LLHR

primer	sequence	comment
BATV L ital 6667 fw	CATAGAGAAGACTATATTCC	endsequencing
BATV L ital 213 rv	TATCTAGAATAATCTCATCC	endsequencing
BATV-Lseg-for	CGGAGTCCCGGGTCAGTAGTGTACTACCGATAC	full genome PCR
BATV-Lseg-rev	CCCAGGTCGGACCGCGAGGAGGTGGAGATGCCATGCCGACCCAGTAGTGTGCTACCGATATAAT	full genome PCR

BATV Tr-for	CCATAGAACAGCATTTAAGATCAATTTCTTTTATATAGGAGCACACTACTGGGTCGGCATGGCATCTCCACC	pHaHdmin amplification
BATV Le-rev	ATTTTATTGATGAATTTTATTGTAATTTCTTTATATAGGAGTACACTACTGACCCGGGACTCCGGGTTTCGTCCTCACGGACTCATCAGAGTAGTGTGCGGCCGCCCTATAGTGAGTCG	pHaHdmin amplification
BATV L 1300 fw	TAATACGTGCCAATCCAC	
BATV L 1401 rv	GCAAATTTATCCAATAGC	
BATV L 1898 fw	GTCAAATTCACTTAAATC	
BATV L 2498 fw	TAACACCTTTAGGGTGGC	
BATV L 3101 fw	AGAGTCTATATGGTTTCC	
BATV L 3605 fw	ACTAACTAAGAAGATACC	
BATV L 4177 fw	TTGCAAATATCCTTGATC	
BATV L 4800 fw	CTCAATTATCTCCCTTTC	Sequencing primer
BATV L 5400 fw	TATAGTAGTATCTTTGAC	
BATV L 6001 fw	ATTTTTGCACTTTAATAC	
BATV L 6600 fw	TTAGATGATAAGTACAAC	
BATV L 7179 fw	GTATCAAAATTGGTAACC	
BATV L 7551 fw	ATGAATAAAGAAGCCAAC	
BATV L rv 4932	GTTTCTCGTTGTTTGTCC	
BATV L 2083 rv	ACAAAACAAGTGCATTGCC	
BATV L 5632 rv	TTTGGTTAGCACTTAGTCC	
